# Serum IGF-1 in patients with rheumatoid arthritis: correlation with disease activity

**DOI:** 10.1186/s13104-022-06008-0

**Published:** 2022-04-05

**Authors:** Hanna Lee, Young Sun Suh, Sang-Il Lee, Yun-Hong Cheon, Mingyo Kim, Hae Sook Noh, Hyun-Ok Kim

**Affiliations:** 1 Division of Rheumatology, Department of Internal Medicine, Gyeongsang National University School of Medicine and Hospital, Jinju, 52727 Korea; 2 Division of Rheumatology, Department of Internal Medicine, Gyeongsang National University School of Medicine and Changwon Hospital, Changwon, 51427 Korea; 3 Division of Rheumatology, Department of Internal Medicine, Gyeongsang National University School of Medicine and Institute of Health Science, Gyeongsang National University Hospital, Jinju, 52727 Korea

**Keywords:** Rheumatoid arthritis, Disease activity, Insulin-like growth factor-1, Insulin-like growth factor binding protein-3

## Abstract

**Objective:**

Insulin-like growth factor (IGF)-1 participates in modulating immunity and inflammation. Its bioactivity is controlled by six IGF-binding proteins (IGFBP-1 to IGFBP-6). In particular, the IGFBP-3 level is reportedly linked to the disease activity of rheumatoid arthritis (RA), consistent with our previous study. Therefore, the present study aimed to reproduce the previous results.

**Results:**

The serum IGFBP-3 level was not significantly different among the three groups according to disease activity based on the DAS28-ESR/CRP (*p* > 0.05) but was significantly different between the low- and high-disease-activity groups based on the DAS28-CRP (*p* = 0.036). Meanwhile, the interleukin-6 (IL-6) level moderately correlated with DAS28-CRP (Spearman’s rho = 0.583, *p* < 0.001).

## Introduction

Rheumatoid arthritis (RA) is a common chronic inflammatory and autoimmune disease primarily involving the synovial membranes and articular structures of multiple joints, although with unknown etiology [1].

In regulating inflammation and immunity, a vast network of interacting cells and cytokines is required*.* The endocrine system also participates in various ways in immune regulation through the actions of growth hormones, especially insulin-like growth factor-1 (IGF-1) [2]. IGF-1 promotes the growth and differentiation of the bone and cartilage tissue and participates in regulating immunity and inflammation [3].

IGF-1 has a fundamental role in both prenatal and postnatal development; it binds to the IGF-1 receptor (IGF-1R) to exert all of its known physiologic effects, which are modulated by multiple IGF-binding proteins (IGFBPs) [4]. Currently, six IGFBP types have been identified (IGFBP-1 to IGFBP-6). Among them, IGFBP-3 is the most important, and it carries up to 75 to 90% of circulating IGF-1 and regulates the action of IGF-1 through interaction with the receptor IGF-1R [5]. Several rheumatic diseases, such as osteoarthritis, diffuse idiopathic skeletal hyperostosis, and RA, exhibit abnormalities in the serum and/or synovial fluid levels of growth hormone (GH)/IGF-1 [3, 6, 7]. However, the mechanism on how the IGF system is involved in RA disease activity remains poorly known. Our previous study showed that the IGFBP-3 levels were significantly higher in patients with RA, particularly in those with active RA. Furthermore, the IGFBP-3 levels in the serum and synovial fluid had a significantly positive correlation with the erythrocyte sedimentation rate (ESR) and CRP level that highly correlate with RA disease activity in patients with RA [8].

However, the serum concentration of IGFBP-3 either increases [9, 10] or decreases [11], depending on the results presented in the previous studies, presumably because of differences in the patient’s age or RA activity level.

Therefore, this study aimed to determine the relationship between the IGFBP-3 system and the disease activity of RA and to reproduce the results of our previous study.

## Main text

### Material and methods

#### Patients

From January 2014 to June 2014, we included 80 patients diagnosed with RA (62 females, 18 males) in the outpatient department of the Division of Rheumatology in Gyeongsang National University Hospital located in Jinju, Korea. RA diagnosis was based on the 1987 and/or 2010 American College of Rheumatology criteria/European League against Rheumatism collaboration. All patients were treated by the same group of rheumatologists.

A serum sample was obtained from each patient and was stored at − 80 ℃ until analysis. The institutional review board of the abovementioned hospital approved this study (2013-03-006), which conformed to the Declaration of Helsinki II. All patients were informed and gave their written consent.

#### Clinical data and measurement of inflammatory markers

All patients were followed up to obtain the following clinical data: age, sex, smoking history, ESR, C-reactive protein (CRP), rheumatoid factor (RF), anti-cyclic citrullinated peptide (anti-CCP) antibody, and the disease activity score in 28 joints (DAS28) with ESR/CRP (DAS28-ESR/CRP).

DAS28 was calculated as described in this link: http://www.4s-dawn.com/DAS28/. We then classified the patients into low-, moderate-, and high-disease-activity groups according to the DAS28 results (low, < 3.2; moderate, 3.2–5.1; high > 5.1) [12].

#### Measurement of IGF-1 and IGFBP3

Serum IGF-1 and IGFBP-3 levels were measured by enzyme-linked immunosorbent assay (ELISA) using a commercial kit (R&D systems) according to the manufacturer’s instructions.

#### Statistical analysis

All statistical data were analyzed using SPSS 21.0 (SPSS Inc., Chicago, IL, USA). In addition, *p* < 0.05 indicated a significant difference. Using the Kruskal–Wallis test, we compared the serum cytokine levels among the patient groups. Furthermore, the correlations of serum cytokine levels with the DAS28-ESR/CRP were investigated by Spearman’s rank correlation analysis.

## Results

### Patient baseline characteristics

Table 1 summarizes the baseline clinical and laboratory characteristics of the 80 study patients. Their mean age was 59.1 ± 9.6 years, and 62 (77.5%) of them were female. Furthermore, 69 (86.3%) were RF positive, and 67 (85.9%) were anti-CCP antibody positive.

**Table 1:** Baseline characteristics of the study patients

Characteristics	Patients (n = 80)
Age, years	59.1 ± 9.6
Gender, male:female	18:62
Rheumatoid factor (positive)	69 (86.3)
Anti-CCP antibody (positive)	67 (83.8)
ESR, mm/h	51.8 ± 32.3
CRP, mg/L	15.4 ± 22.9
Pain (VAS), mm	3.5 ± 2.7
Tender joint count	4.8 ± 5.2
Swollen joint count	5.7 ± 6.0
DAS28-ESR	4.7 ± 1.9
DAS28-CRP	3.8 ± 1.8
IGF-1, pg/ml	286.2 ± 264.5
IGFBP-3, pg/ml	5581.1 ± 1172.5
TNF-α, pg/ml IL-1, pg/ml IL-6, pg/ml	38.2 ± 113.75 6.4 ± 16.40 17.2 ± 30.97

Numeral data are presented as mean ± one standard deviation, or number of patients with percentages in parentheses, as appropriate

*Anti-CCP* anti-cyclic citrullinated peptide, *CRP* C-reactive protein, *DAS* disease activity score, *ESR* estimated sedimentation rate, *IGF* insulin-like growth factor, *IGFBP* insulin-like growth factor binding protein, *IL* interleukin, *TNF-α* tumor necrosis factor alpha, *VAS* visual analog scale

### Relationship between serum cytokines and disease activity based on DAS28 ESR

According to the DAS28-ESR results, 20, 21, and 39 patients were categorized into the low-, moderate-, and high-disease-activity groups, respectively. Among these three groups, no significant difference was observed in terms of age and gender (*p* > 0.05) and the IGFBP**-**3 level (Table 2, *p* > 0.05). In addition, no significant difference was found between the IGFBP**-**3 or IGF-1 level and ESR (*p* > 0.05). However, the IL (interleukin)-6 level was significantly different among the three groups (Table 2, *p* < 0.001) but moderately correlated with the DAS28-ESR (Spearman’s rho = 0.602, *p* < 0.001).

**Table 2:** Comparison of serum cytokines among patient groups according to disease activity based on DAS28-ESR

Serum cytokines	Low disease activity (n = 20)	Moderate disease activity (n = 21)	High disease activity (n = 39)	*p* value
IGF-1 (pg/ml)	166.200 (116.305, 307.555)	153.070 (100.038, 327.442)	229.920 (142.760, 423.772)	0.314
IGFBP-3 (pg/ml)	5359.140 (5001.525, 5901.715)	5818.650 (5105.312, 6321.658)	5732.090 (5277.685, 6779.132)	0.175
TNF-α (pg/ml)	6.545	7.661	9.680	0.650
	(3.412, 13.093)	(4.109, 12.989)	(4.736, 13.789)	
IL-1 (pg/ml)	2.189	2.262	2.557	0.270
	(1.630, 2.939)	(2.066, 3.120)	(2.139, 3.092)	
IL-6 (pg/ml)	1.819 (0.809, 2.588)	4.569 (2.871, 11.973)	13.399 (3.720, 36.984)	< 0.001

Numeral data are presented as mean ± one standard deviation, or number of patients with percentages in parentheses, as appropriate

DAS28-*ESR* disease activity score in 28 joints with estimated sedimentation rate, *IGF* insulin-like growth factor, *IGFBP* insulin-like factor binding protein, *IL* interleukin, *TNF-α* tumor necrosis factor alpha

### Relationship between serum cytokines and disease activity based on DAS28-CRP

According to the DAS28-CRP results, 35, 23, and 22 patients were categorized into the low-, moderate-, and high-disease-activity groups, respectively. Among these groups, no significant difference was found in terms of age and gender (*p* > 0.05), the IGFBP-3 level (Table 3, *p* > 0.05), and the IL-6 level (Table 3, *p* < 0.001). However, the IGF**-**1 level was significantly different between the low- and high-disease-activity groups (*p* = 0.036). Meanwhile, no significant difference was found between the IGFBP**-**3 or IGF-1 level and CRP (*p* > 0.05). Additionally, the IL-6 level moderately correlated with DAS28-CRP (Spearman’s rho = 0.583, *p* < 0.001).

**Table 3:** Comparison of serum cytokines among patient groups according to disease activity based on DAS28-CRP

Serum cytokines	Low disease activity (n = 35)	Moderate disease activity (n = 23)	High disease activity (n = 22)	*p* value
IGF-1 (pg/ml)	153.070 (98.480, 311.705)	221.250 (110.138, 470.130)	245.990 (191.280, 401.430)	0.036
IGFBP-3 (pg/ml)	5563.720 (5068.687, 6101.353)	5732.090 (5286.497, 6142.102)	5706.875 (5227.440, 6868.740)	0.587
TNF-α (pg/ml)	5.989 (2.821, 13.163)	7.800 (4.736, 12.814)	10.098 (4.945, 13.580)	0.425
IL-1 (pg/ml)	2.189 (1.844, 2.951)	2.410 (2.016, 3.074)	2.557 (2.167, 3.086)	0.167
IL-6 (pg/ml)	2.547 (1.739, 4.286)	4.447 (3.578, 15.119)	33.513 (6.752, 58.346)	< 0.001

Numeral data are presented as mean ± one standard deviation, or number of patients with percentages in parentheses as appropriates

*DAS28-CRP* disease activity score in 28 joints with C-reactive protein, *IGF* insulin-like growth factor, *IGFBP* insulin-like factor binding protein, *IL* interleukin, *TNF-α* tumor necrosis factor alpha

## Discussion

As a lymphohemopoietic cytokine, IGF-1 has profound positive effects on immune function [13]. IGF-1 binds to T and B cells [14] and induces anti-CD3-stimulated T cell proliferation and chemotaxis [13, 15]. It also activates T cell Akt, thereby enhancing lymphocyte survival [16]. Recently, IGF-1 reportedly prevents cord blood T cells from spontaneous apoptosis when cultured in a serum-free medium. Furthermore, IGF-1 significantly inhibits the spontaneous apoptosis of all the subsets of cord blood T cells [17]. Hence, IGF-1 influences the onset of inflammatory diseases, thereby may be a useful target. The abundance and profile of IGFBPs serve as important determinants of signaling by influencing IGF availability for receptor binding [13]. Thus, our group previously confirmed the correlation between IGFBP-3, which is essential in IGF-1 activity, and RA activity. Previously, we showed that the IGFBP-3 levels in the serum and synovial fluid were significantly higher in patients with RA and had a significantly positive correlation with the ESR and CRP levels that highly correlate with RA disease activity in these patients [8]. However, ESR and CRP have limitations in reflecting clinical symptoms such as joint swelling or tenderness when evaluating disease activity of patients with RA. Therefore, in the present study, we investigated patient’s disease activity and IGFBP-3 levels by using DAS28-ESR/CRP, which is used to measure disease activity in patients with RA. Contrary to our hypothesis, DAS28-ESR/CRP and IGFBP-3 showed no significant association. Unlike the previous results, IGFPB3 had no correlation with ESR and CRP. Thus, in this study, we investigated the correlation between DAS28-ESR/CRP and IGF-1, which is the major ligand for IGFBP-3, in patients with RA. Interestingly, IGF-1 reflects a statistically significant difference between the high-disease-activity and low-disease-activity groups of DAS28-CRP. However, IGF-1 had no correlation with ESR and CRP. Moreover, IL-6 was statistically significant in reflecting the RA activity. IL-6 is one of the cytokines that play an important role in RA pathogenesis. In an animal study, IL-6 functioned as an antiapoptotic cytokine that prevents T cell apoptosis [18]. IL-6 promotes B cell differentiation into the plasma cell and also influences T cell development by stimulating T cell proliferation and differentiation into Th-17 cells, which produce IL-17 [19]. In the current study, IGF-1 and IL-6 reflected RA activity through the use of DAS28-CRP, but the results of this study alone limitedly explained the interaction between IGF-1 and IL-6. Nevertheless, on the basis of the results of existing research, our results allow to hypothesize that IGF-1 may prevent T cell apoptosis and contribute to the antiapoptotic effect in part via IL-6. Further investigation is required to elucidate the correlation of IL-6 and IGF-1 with the RA activity.

## Limitations

Our study has several possible limitations. First, we had no control group to be compared with our patients with RA. Second, the evaluation method of RA disease activity is inadequate. We only used the DAS28-ESR/CRP to evaluate the RA disease activity. According to a recent study [20], clinical disease activity index and simplified disease activity index showed more congruous classification of disease activity than DAS28-ESR across different populations. Therefore, further study is necessary to confirm our results with the evaluation of these indices.

## Data Availability

All data generated or analyzed during this study are included in this published article.

## Abbreviations

RA: Rheumatoid arthritis
IGF: Insulin-like growth factor
IGFBP: Insulin-like growth factor binding protein
